# Dysregulated microRNAs in Hepatitis B Virus-Related Hepatocellular Carcinoma: Potential as Biomarkers and Therapeutic Targets

**DOI:** 10.3389/fonc.2020.01271

**Published:** 2020-07-28

**Authors:** Jinghang Xu, Ping An, Cheryl A. Winkler, Yanyan Yu

**Affiliations:** ^1^Department of Infectious Diseases, Center for Liver Diseases, Peking University First Hospital, Peking University, Beijing, China; ^2^Basic Research Laboratory, Molecular Genetic Epidemiology Section, Basic Science Program, Frederick National Laboratory for Cancer Research, Frederick, MD, United States

**Keywords:** biomarkers, hepatitis B virus, hepatocellular carcinoma, microRNA, gene expression, early diagnosis, prognosis

## Abstract

MicroRNAs (miRNAs) are non-coding small RNAs that can function as gene regulators and are involved in tumorigenesis. We review the commonly dysregulated miRNAs in liver tumor tissues and plasma/serum of hepatitis B virus (HBV)-related hepatocellular carcinoma (HCC) patients. The frequently reported up-regulated miRNAs in liver tumor tissues include miR-18a, miR-21, miR-221, miR-222, and miR-224, whereas down-regulated miRNAs include miR-26a, miR-101, miR-122, miR-125b, miR-145, miR-199a, miR-199b, miR-200a, and miR-223. For a subset of these miRNAs (up-regulated miR-222 and miR-224, down-regulated miR-26a and miR-125b), the pattern of dysregulated circulating miRNAs in plasma/serum is mirrored in tumor tissue based on multiple independent studies. Dysregulated miRNAs target oncogenes or tumor suppressor genes involved in hepatocarcinogenesis. Normalization of dysregulated miRNAs by up- or down-regulation has been shown to inhibit HCC cell proliferation or sensitize liver cancer cells to chemotherapeutic treatment. miRNAs hold as yet unrealized potential as biomarkers for early detection of HCC and as precision therapeutic targets, but further studies in diverse populations and across all stages of HCC are needed.

## Introduction

Hepatocellular carcinoma (HCC) is one of the most common and deadly cancers in the world (1, 2). Major risk factors for HCC are chronic infection by hepatitis B virus (HBV) or hepatitis C virus (HCV) (3). HCC is usually diagnosed at the late stages, due to the low sensitivity of the current diagnostic methods, which include imaging and quantification of alpha-fetoprotein (AFP) levels. Although recent advances in genomic technology have identified a variety of genetic alterations in HCC tissues, convenient biomarkers with sufficient sensitivity and specificity for early diagnosis of HCC are still lacking.

Detection of microRNAs (miRNAs) has recently gained increasing attention for their potential utility in the early diagnosis of HCC. miRNAs are one of the major post-transcriptional regulators of gene expression. As non-coding small endogenous RNAs with ~22 nucleotides, miRNAs silence genes by binding to the 3' untranslated region (3' UTR) of messenger RNAs (mRNAs) and triggering mRNA degradation or translational repression (4–6). To date, more than 2,600 mature human miRNAs have been listed on the miRbase database (http://www.mirbase.org). Each miRNA can target multiple mRNAs with varying effects and a single mRNA may be targeted by multiple miRNAs. miRNAs modulate various biological molecular pathways and cellular processes, including cell proliferation, differentiation, development, apoptosis, angiogenesis, metabolism, and immune responses (7–10). Dysregulated miRNAs have been implicated in the development of a variety of tumors, including HCC, and may serve as robust biomarkers for cancer diagnosis and prognosis (11–14).

**Figure 1:**
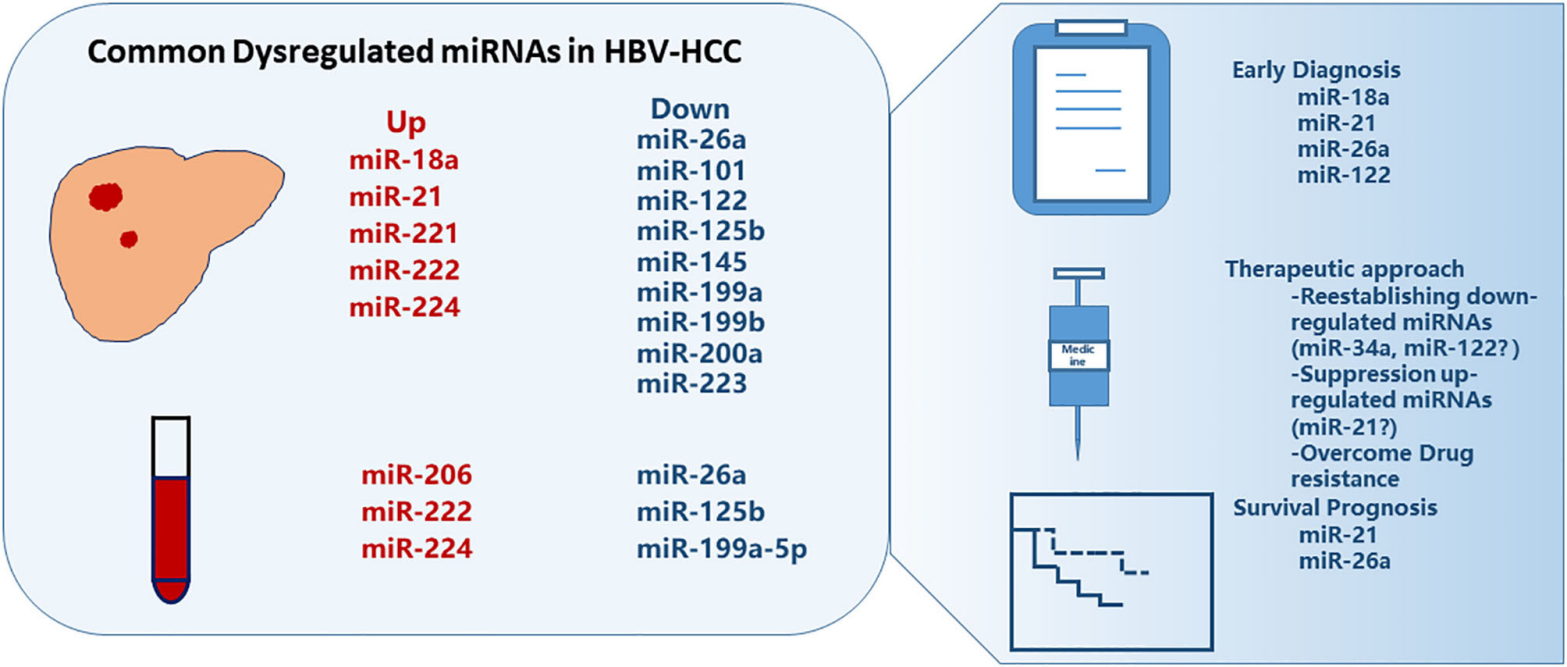
Commonly dysregulated microRNAs in HBV-related HCC. Several miRNAs are up- or down-regulated in liver tumor tissues or in plasma/serum, some of which showed promise for early diagnosis and survival prognosis of HCC, and can be manipulated for treatment.

Given that miRNAs expression levels might differ among HCC patients with different etiological factors (15) and that HBV is the predominant risk factor for HCC (16), the present review focuses on miRNAs involved with HBV-related HCC (HBV-HCC). We have assessed patterns of reported dysregulated miRNAs in the HBV-HCC patients and present the mechanisms and potential applications of miRNAs in the diagnosis, prognosis, and treatment of HBV-HCC (Figure 1).

## Dysregulated miRNAs IN HBV-HCC

Comparisons of HBV-HCC tumor tissue to either matched non-tumor tissue or liver tissue from healthy controls indicate that a subset of miRNAs is differentially expressed between health and tumor tissues. In Table 1, we list miRNAs that have been replicated in at least two HBV-HCC studies. Commonly reported up-regulated miRNAs include miR-18a, miR-21, miR-221, miR-222, and miR-224, whereas down-regulated miRNAs include miR-26a, miR-101, miR-122, miR-125b, miR-145, miR-199a, miR-199b, miR-200a, and miR-223 (17–36).

**Table 1:** Dysregulated microRNAs in the tissue of predominantly HBV-related HCC.

**miRNAs**	**Dysregulation** **type**	**Fold change**	**Case vs. control**	**Samples details**		**References**
				**Size and HBV status**	**Underlying cirrhosis % (*n*)**	
miR-18a	Up-regulated	0.585^a^	HCC vs. ANT	78 HCC (62 HBV)	51% (40/78)	(17)
	Up-regulated	3.223^b^	HCC vs. ANT	22 HCC (20 HBV)	NA	(18)
miR-21	Up-regulated	2.29^a^	HCC vs. ANT	100 HCC (58 HBV, 8 HCV, 27 NBNC, 27 Unknown)	46% (46/100)	(19)
	Up-regulated	3.67^b^	HCC vs. ANT	115 HCC (101 HBV)	51% (59/115)	(20)
	Up-regulated	NA	HCC vs. ANT	148 HCC (82 HBV)	41% (45/109)	(21)
	Up-regulated	NA	HCC vs. ANT	31 HBV-HCC	NA	(22)
	Up-regulated	NA	HCC vs. ANT	24 HBV-DNs, 29 small HBV-HCC nodules, 38 HBV-ANTs	92% (22/24) in DNs 93% (27/29) in HCC	(23)
	Up-regulated	3.72^b^	HCC vs. ANT	42 HBV-HCC	NA	(24)
miR-221	Up-regulated	1.51^a^	HCC vs. ANT	100 HCC (58 HBV, 8 HCV, 27 NBNC, 27 Unknown)	46% (46/100)	(19)
	Up-regulated	NA	HCC vs. ANT	135 HCC (96 HBV)	95% (128/135)	(25)
	Up-regulated	4.00^b^	HCC vs. ANT	115 HCC (101 HBV)	51% (59/115)	(20)
	Up-regulated	NA	HCC vs. ANT	31 HBV-HCC	NA	(22)
	Up-regulated	NA	HCC vs. ANT	24 HBV-DNs, 29 small HBV-HCC Nodules, 38 HBV-ANTs	92% (22/24) in DNs 93% (27/29) in HCC	(23)
	Up-regulated	1.57^a^	HCC vs. ANT	78 HCC (62 HBV)	51% (40/78)	(17)
miR-222	Up-regulated	1.41^a^	HCC vs. ANT	78 HCC (62 HBV)	51% (40/78)	(17)
	Up-regulated	4.44^b^	HCC vs. ANT	115 HCC (101 HBV)	51% (59/115)	(20)
	Up-regulated	4.964^b^	HCC vs. ANT	22 HCC (20 HBV)	NA	(18)
	Up-regulated	NA	HCC vs. ANT	42 HCC (33 HBV), 6 HCV, 3 NBNC-HCC	85% (28/33)	(26)
miR-224	Up-regulated	NA	HCC vs. ANT	24 HBV-DNs, 29 small HBV-HCC Nodules, 38 HBV-ANTs	92% (22/24) in DNs 93% (27/29) in HCC	(23)
	Up-regulated	27.231^b^	HCC vs. ANT	22 HCC (20 HBV)	NA	(18)
	Up-regulated	0.903^a^	HCC vs. ANT	78 HCC (62 HBV)	51% (40/78)	(17)
miR-26a	Down-regulated	0.37 ^b^	HCC vs. ANT	455 HCC (412 HBV)	88% (400/455)	(27)
	Down-regulated	−1.59^a^	HCC vs. ANT	100 HCC (58 HBV, 8 HCV, 27 NBNC, 27 Unknown)	46% (46/100)	(19)
miR-101	Down-regulated	NA	HCC vs. ANT	25 HCC (20 HBV), 20 HC (HBV negative)	72% (18/25)	(28)
	Down-regulated	NA	HCC vs. HC HCC vs. CHB HCC vs. LC	67 HBV-HCC, 61 HBV-LC, 79 CHB, 30 Normal control	NA	(29)
	Down-regulated	0.214^b^	HCC vs. ANT	22 HCC (20 HBV)	NA	(18)
	Down-regulated	−0.958^a^	HCC vs. ANT	78 HCC (62 HBV)	51% (40/78)	(17)
miR-122	Down-regulated	−1.67^a^	HCC vs. ANT	100 HCC (58 HBV, 8 HCV, 27 NBNC, 27 Unknown)	46% (46/100)	(19)
	Down-regulated	NA	HCC vs. ANT	97 HCC (84 HBV)	NA	(30)
	Down-regulated	NA	HCC vs. ANT, HBV-HCC vs. non-HBV-HCC	142 HCC (103 HBV)	58% (82/142)	(31)
	Down-regulated	NA	HCC vs. ANT	24 HBV-DNs, 29 small HBV-HCC Nodules, 38 HBV-ANTs	92% (22/24) in DNs 93% (27/29) in HCC	(23)
	Down-regulated	0.60^b^	Venous metastases vs. solitary tumors	214 HBV-HCC	93% (199/214)	(32)
miR-125b	Down-regulated	−0.893^a^	HCC vs. ANT	78 HCC (62 HBV)	51% (40/78)	(17)
	Down-regulated	NA	HCC vs. ANT	97 HCC (84 HBV)	NA	(30)
	Down-regulated	0.58^b^	Venous metastases vs. solitary tumors	214 HBV-HCC	93% (199/214)	(32)
miR-145	Down-regulated	NA	HCC vs. ANT LGDN vs. ANT HGDN vs. ANT	24 HBV-DNs, 29 small HBV-HCC Nodules, 38 HBV-ANTs	92% (22/24) in DNs 93% (27/29) in HCC	(23)
		0.28^b^	HCC vs. ANT	42 HBV-HCC	NA	(24)
miR-145-5P	Down-Regulated	−2.39^a^	HCC vs. ANT	100 HCC (58 HBV, 8 HCV, 27 NBNC, 27 Unknown)	46% (46/100)	(19)
miR-199a	Down-regulated	0.149^b^	HCC vs. ANT	22 HCC (20 HBV)	NA	(18)
	Down-regulated	NA	HCC vs. ANT	97 HCC (84 HBV)	NA	(30)
miR-199a-5P	Down-regulated	−4.51^a^	HCC vs. ANT	100 HCC (58 HBV, 8 HCV, 27 NBNC, 27 Unknown)	46% (46/100)	(19)
miR-199a-3P	Down-regulated	−2.78^a^	HCC vs. ANT	100 HCC (58 HBV, 8 HCV, 27 NBNC, 27 Unknown)	46% (46/100)	(19)
miR-199b	Down-regulated	NA	HCC vs. ANT LGDN vs. ANT HGDN vs. ANT	24 HBV-DNs, 29 small HBV-HCC Nodules, 38 HBV-ANTs	92% (22/24) in DNs 93% (27/29) in HCC	(23)
miR-200a	Down-regulated	NA	HCC vs. ANT	120 HCC (97 HBV)	78% (93/120)	(33)
	Down-regulated	0.421^b^	HCC vs. ANT	101 HCC (71 HBV)	NA	(34)
	Down-regulated	0.522^b^	HCC vs. ANT	95 HCC (78 HBV)	47% (45/95)	(35)
miR-223	Down-regulated	−1.92^a^	HCC vs. ANT	100 HCC (58 HBV)	46% (46/100)	(19)
	Down-regulated	0.267^b^	HCC vs. ANT	22 HCC (20 HBV)	NA	(18)
	Down-regulated	0.20^b^	HCC vs. ANT	42 HCC (33 HBV), 6 HCV, 3 NBNC-HCC	85% (28/33)	(26)

Fold changes were based on the original report;

a Log_2_ fold change.

b Regular fold change.

*ANT, adjacent non-cancerous tissue; CHB, chronic hepatitis B; DN, dysplastic nodules; HBV, hepatitis B virus; HC, healthy control; HCC, hepatocellular carcinoma; HCV, hepatitis C virus; HGDN, high-grade dysplastic nodule; LC, liver cirrhosis; LGDN, low-grade dysplastic nodule; NA, not available; NBNC, non-HBV non-HCV*.

Due to limited liver tissue accessibility and the invasive nature of biopsy, studies assessing circulating miRNAs in plasma or serum from patients with HBV-HCC have increased dramatically in recent years. Cellular miRNAs from tumors leak into the circulation system following cell injury, apoptosis, and necrosis or by secretion through cell-derived exosomes and shedding vesicles (37). Circulating miRNAs in serum or plasma are stable (38), suggesting that circulating miRNAs may be accessible and quantifiable cancer diagnostic or prognostic biomarkers. Commonly reported dysregulated circulating miRNAs from patients with HBV-HCC include miR-21, miR-26, miR-122, miR-125b, miR-192, miR-206, miR-222, miR-223, and miR-224 (28, 29, 39–46) (Table 2).

**Table 2:** Dysregulated microRNAs in the plasma/serum of patients with HBV-related HCC.

**miRNAs**	**Dysregulation type**	**Fold change**	**Case vs. control**	**Samples details**	**References**
miR-18a	Up-regulated	NA	HCC vs. HC, HCC vs. (CHB + LC)	101 HBV-HCC, 30 CHB or HBV-LC, 60 HC	(44)
miR-192	Up-regulated	1.4^b^	HCC vs. (LC+CHB+HC)	457 HBV-HCC, 141 HBV-LC, 169 CHB, 167 HC	(45)
miR-206	Up-regulated	9.94^b^	HCC vs. HC	261 HBV-HCC, 173 HC	(46)
	Up-regulated	3.51^b^	HCC vs. LC	261 HBV-HCC, 233 HBV-LC	(46)
	Up-regulated	2.98 ± 3.94^b^	HCC vs. matched control	55 HBV-HCC, 50 age and gender-matched control	(39)
miR-221	Up-regulated	4.83^b^	HCC vs. HC	46 HCC (30 HBV), 20 HC	(41)
miR-222	Up-regulated	NA	HCC vs. HC	70 HBV-HCC, 48 CHB, 34 HC	(43)
	Up-regulated	3.01^b^	HCC vs. HC	46 HCC (30 HBV), 20 HC	(41)
miR-224	Up-regulated	1.88^b^	HCC vs. HC	46 HCC (30 HBV), 20 HC	(41)
miR-21	Up-regulated	1.9^b^	HCC vs. (LC+CHB+HC)	457 HBV-HCC, 141 HBV-LC, 169 CHB, 167 HC	(45)
	Up-regulated	NA	HCC vs. HC	97 HCC (60 HBV), 30 HC	(47)
	Up-regulated	2.85^b^	HCC vs. HC	46 HCC (30 HBV), 20 HC	(41)
	Up-regulated	NA	HCC vs. HC	101 HCC (76 HBV), 48 CHB, 89 HC	(42)
	Down-regulated	NA	HCC vs. CHB	101 HCC (76 HBV), 48 CHB, 89 HC	(42)
miR-122	Up-regulated	4.09 ± 5.38 ^b^	HCC vs. HBV (ASC +CHB)	65 HBV-HCC, 160 controls	(39)
	Up-regulated	NA	HCC vs. HC	70 HBV-HCC, 48 CHB, 34 HC	(43)
	Up-regulated	NA	HCC vs. HC	101 HCC (76 HBV), 48 CHB, 89 HC	(42)
	Down-regulated	0.7^b^	HCC vs. (LC+CHB+HC)	457 HBV-HCC, 141 HBV-LC, 169 CHB, 167 HC	(45)
	Down-regulated	NA	HCC vs. CHB	101 HCC (76 HBV), 48 CHB, 89 HC	(42)
miR-192-5p	Up-regulated	1.71^b^	HCC vs. HC	212 HBV-HCC, 110 HC	(48)
	Up-regulated	1.97^b^	HCC vs. LC	212 HBV-HCC, 106 HBV-LC	(48)
	Down-regulated	0.85^b^	HCC vs. LC	261 HBV-HCC, 233 HBV-LC	(46)
	Down-regulated	0.77^b^	HCC vs. HC	261 HBV-HCC, 173 HC	(46)
miR-223	Up-regulated	NA	HCC vs. HC	101 HCC (76 HBV), 89 HC	(42)
	Up-regulated	2.97 ± 1.67 ^b^	HCC vs. HC	65 HBV-HCC, 160 controls	(39)
	Up-regulated	NA	HCC vs. HC	70 HBV-HCC, 34 HC	(43)
	No difference	NA	HCC vs. CHB	101 HCC (76 HBV), 48 CHB	(42)
	Down-regulated	0.3^b^	HCC vs. (LC+CHB+HC)	457 HBV-HCC, 141 HBV-LC, 169 CHB, 167 HC	(45)
	Down-regulated	NA	HCC vs. HBV (ASC +CHB)	65 HBV-HCC, 135 HBV (55 ASC+ 80 CHB)	(39)
miR-26a	Down-regulated	0.2^b^	HCC vs. (LC+CHB+HC)	457 HBV-HCC, 141 HBV-LC, 169 CHB, 167 HC	(45)
miR-26a-5p	Down-regulated	0.65^b^	HCC vs. HC	261 HBV-HCC, 173 HC	(46)
	Down-regulated	0.54^b^	HCC vs. LC	261 HBV-HCC, 233 HBV-LC	(46)
miR-122-5p	Down-regulated	0.27^b^	HCC vs. HC	261 HBV-HCC, 173 HC	(46)
	Down-regulated	0.54^b^	HCC vs. LC	261 HBV-HCC, 233 HBV-LC	(46)
miR-125b	Down-regulated	0.26 ± 0.46^b^	HCC vs. HBV (ASC +CHB)	65 HBV-HCC, 135 HBV (55 ASC+ 80 CHB)	(39)
	Down-regulated	NA	HCC vs. LC	30 HCC (28 HBV), 30 LC (27 HBV)	(40)
	Down-regulated	NA	HCC vs. CHB	30 HCC (28 HBV), 30 CHB	(40)
miR-199a-5p	Down-regulated	0.58^b^	HCC vs. HC	261 HBV-HCC, 173 HC	(46)
	Down-regulated	0.87^b^	HCC vs. LC	261 HBV-HCC, 233 HBV-LC	(46)

Fold changes were based on the original report;

a Log_2_ fold change.

b Regular fold change.

*ASC, asymptomatic carrier; CHB, chronic hepatitis B; HBV, Hepatitis B virus; HBV-HCC, HBV-related HCC; HBV-LC, HBV-related LC; HC, healthy control; HCC, hepatocellular carcinoma; LC, liver cirrhosis; NA, not available*.

For a subset of the miRNAs [e.g., up-regulated miR-18a, miR-221, miR-222, miR-224, and down-regulated miR-26a and miR-125b (Table 3)], dysregulated patterns were consistent among multiple independent studies and between tumor tissue and serum/plasma. These microRNAs may be of more translational value in the diagnosis, differential diagnosis or even therapy for HBV-HCC. However, other miRNAs showed inconsistent or contrasting profiles of dysregulation among studies or between tumor tissue and serum/plasma (Tables 1, 2). For example, downregulation of miR-122 was common in HCC tissue (19, 23, 32), but circulating miRNA levels were upregulated in some studies (39, 42, 43) and downregulated in others (45). Based on the observation that increased serum miR-122 is presented in both HCC patients and chronic hepatitis patients, some researchers speculate that higher levels of miR-122 in serum may result from liver injury rather than HCC itself (42, 43). It is also likely that factors governing the expression of miRNAs in the tissues and sera of HCC patients might differ. Additional factors that may contribute to discordant findings among these results include differences in patient selection, tumor stage, biological sample handling, and storage, miRNA probes employed, sample size, or genetic background of study populations (49).

**Table 3:** Common consistently dysregulated microRNAs between tumor tissue and serum/plasma in HBV- HCC.

**Dysregulation type**	**miRNAs**	Publication numbers^*^ (in tissue)	Publication numbers^*^ (in serum/plasma)
Up-regulated	miR-18a	2	1
	miR-221	6	1
	miR-222	4	2
	miR-224	3	1
Down-regulated	miR-26a	2	1
	miR- 125b	3	3

* *Publications cited in Table 1 and Table 2. Results in tissue and serum/plasma don't necessarily origin from the same study*.

## Mechanism of miRNA Dysregulation IN HCC

**Figure 2:**
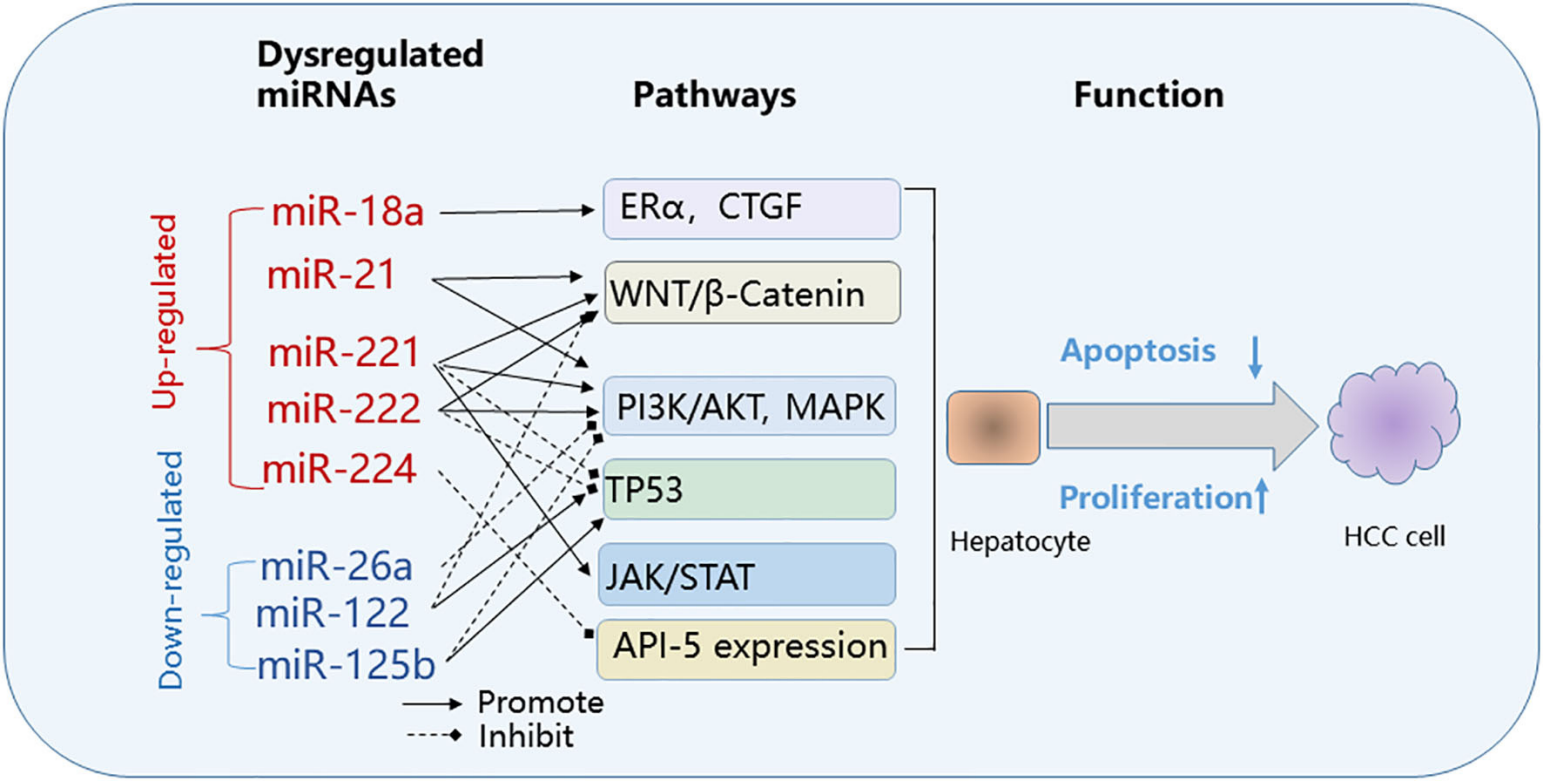
Common pathways targeted by dysregulated microRNAs in HBV-related HCC. Effect on cancer pathways by dysregulated miRNAs: upregulated (red); down-regulated (blue). API-5, apoptosis inhibitor-5; CTGF, connective tissue growth factor; ERα, estrogen receptorα; HBV, hepatitis B virus; HCC, hepatocellular carcinoma; JAK/STAT, Janus kinase/signal transducer; PI3K/MAPK, phosphoinositide 3-kinase/mitogen-activated protein kinase; WNT/β-Catenin, wingless-related integration site/beta-catenin.

It's not fully understood if miRNA dysregulation in HCC is the cause, consequence of HCC development or both. Accumulating evidence indicates that some dysregulated miRNAs are active players in tumor initiation and progression. The direct targets of miRNAs may be protein-coding genes involved in any or all pathophysiological mechanisms of cancer development, including cell growth, apoptosis, invasion, and metastasis. miRNAs may function as either tumor promoters or tumor suppressors depending on their target genes (50). miRNAs in HCC that target and suppress oncogenes may be down-regulated, while miRNAs that target suppressor genes may be up-regulated during tumor development (Figure 2). The miR-122 expression is largely liver-specific and under transcriptional control by the liver-enriched transcription factors HNF1A, HNF3A, and HNF3B (51). miR-122 can function as a tumor suppressor by suppressing HCC growth, invasion, migration, angiogenesis and by increasing HCC apoptosis and cell cycle arrest (52). miRNA-122 targets multiple genes, including *BCL9, Bcl-w, NDRG3*, cyclin G1, *ADAM17, ADAM10, G6PD*, and pituitary tumor-transforming gene 1 (*PTTG1*) binding factor (*PBF*), all of which have been implicated in tumor development (53–60). Other miRNAs such as miRNA-21 function as oncogenes by stimulating HCC growth, invasion, and migration (23, 61, 62). The inhibition of miR-21 suppresses HCC tumor growth (63).

Dysregulated miRNAs affect key cellular pathways that play a role in the pathogenesis of HBV-HCC (Figure 2). The commonly targeted pathways by dysregulated miRNAs in HBV-HCC include the Janus kinase/signal transducer (JAK/STAT), phosphoinositide 3-kinase/mitogen-activated protein kinase (PI3K)/AKT and MAPK, Wingless-related integration site/beta-catenin (WNT/β-Catenin) and TP-53 pathways (40, 64–73).

### Interaction of miRNAs and HBV in HBV-HCC

HBV can directly regulate cellular miRNAs levels. miR-122 is targeted and inhibited by HBV mRNA, which harbors a miR-122 complementary site, leading to the upregulation of the PTTG1-binding protein and promotion of HCC tumor growth and cell invasion (57). Down-regulation of miR-122 occurs mainly in HBV-HCCs but not in HCV-infected HCCs (74). HBV downregulates miR-101 expression by directly inhibiting its promoter activity (75). Hepatitis B X antigen (HBx) increases the expression of miR-21 and subsequently promotes the progression of HCC by targeting *PTEN* and the tumor suppressor *PDCD4* (61). HBx suppresses p53-mediated activation of miR-148a thereby promoting tumor growth and metastasis; expression of miR-148a reduced tumor growth and invasion. In patients with HBV-HCC, miR-148a was down-regulated. These results suggest that activation of miRNA-148a or down-regulation of its targeted pathways may have a role in HCC treatment (76).

In contrast, cellular miRNAs, including miR-122, and miR-125 and miR-199 family members, affect HBV replication (77). miR-125a-5p, markedly downregulated in HCC, inhibits HBsAg expression and secretion (78).

Using RNA deep sequencing and northern blotting, HBV-encoded miRNAs were recently identified. HBV-miR-3 was shown to restrict HBV replication, by targeting the region of HBV 3.5-kb mRNA encoding HBV core antigen (HBc) (79). Another HBV-encoded miRNA, HBV-miR-2, can promote the oncogenic activity of liver cancer cells (80). HBV-encoded miRNAs likely contribute to HBV-specific HCC development.

The complex interactions and molecular interactions among cellular miRNAs and HBV have been reviewed in (73, 81–83).

### Dysregulated miRNAs in Liver Cancer Stem Cells (LCSCs)

Cancer stem cells are small subpopulations of tumor-initiating cells within tumors that capable of self-renewal, differentiation, and proliferation. LCSCs can be identified by several highly expressed stem cell surface markers including epithelial cell adhesion molecule (EpCAM), CD90, CD44, CD133, and CD13 (84, 85). The other reported LCSC surface markers include OV6, DLK1, ABCG2, ALDH, and CD24 (84–87). LCSCs are responsible for tumor initiation, metastasis, relapse, and chemo- and radiation-therapy resistance in liver cancer (87). The specific influence of HBV on LCSCs remains largely unknown. Liver inflammatory damage induced by chronic HBV and HCV infection and liver toxins can induce somatic mutations, genomic instability, and epigenetic perturbations, resulting in the deregulation of self-renewal and differentiation signaling pathways of activated liver progenitor cells, which promotes the transformation of liver progenitor cells into LCSCs (84). It has been reported that HBx promotes the stem-like properties of OV6^+^ CSCs in HBV-related HCC via MDM2 independent of p53 (88). Concomitant elevated expression of HBx and OV6 predicts a poor prognosis for patients with HBV- HCC (88).

Multiple miRNAs have been reported to regulate a variety of biological behaviors of LCSCs, including let-7, miR-200, miR-122, miR-181, miR-1246, miR-152, miR-145, miR-217, miR-500a-3p, and miR-148 (87). miRNAs affect the CSC phenotype by regulating the expression of oncogenes and stem cell-related genes (85). These miRNAs target key molecules in the following pathways involved in carcinogenesis: *Wnt*/beta-catenin signaling, TGF-beta signaling, JAK/STAT signaling, epithelial-mesenchymal transition (EMT) in LCSCs (87). miRNA profiling comparisons between CSC^+^ and CSC^−^ HCCs, as separated by hepatic CSC biomarkers (EpCAM, CD133, CD90, CD44, and CD24), identified aberrant downregulation of liver-specific miR-192-5p in HCC cells, which correlated with increased CSC populations with stemness features and shorter survival in HCC patients (89). Over-expression of miR-192-5p inhibited the stemness features of human liver cancer cell lines, with decreased spheroid formation, decreased CSC number and diversity and decreased expression of CSC biomarkers and increased expression of genes related to hepatocyte metabolism (89). Hepatitis B virus X protein (HBx) induces expression of EpCAM by upregulating miR-181 to promote stemness in hepatocarcinogenesis (90, 91). The knockdown of miR-181 significantly reduces the EpCAM^+^ LCSCs and tumor-initiating ability (92).

Targeting the regulation of these miRNAs or their pathways may serve as a potential therapeutic strategy to inhibit or eradicate LCSCs (87). Restoring of miR-122 has been demonstrated to suppresses stem-like HCC cells (93). It would be interesting to explore the clinical utility of restoring the miR-192-5p for riding of LCSCs (89).

### Epigenetic Alterations and miRNAs in HCC

Epigenetic alterations such as DNA methylation and histone modification are essential for chromatin remodeling and regulation of both coding genes and miRNAs. Abnormal DNA methylation patterns of a number of miRNAs in HCC have been reported for hypermethylation of miR-1, miR-9, miR-10a, miR-10b, miR-124, miR-125b, miR-132, miR-148a, miR-195, miR-196b, miR-203, miR-320, miR-375, miR-378, miR-497, miR-596, miR-663, and miR-1247, and for hypomethylation of miR-23a, miR-25, miR-27a, miR-93, and miR-106b (94). Among these miRNAs, only miR-125b presents consistent dysregulation pattern of expression, and was down-regulated both in tissue (17, 30, 32) and serum (39, 40) of patients with HCC. The expression of miR-125b was significantly increased by the methylation inhibitor 5-aza-2'-deoxycytidine in HCC cells, suggesting the epigenetically modulation of the expression of miR-125b (95).

Histone modifications, including acetylation, methylation, and phosphorylation of lysine residues, play an important role in expression regulation of genes including miRNAs in HCC tumor tissue. For example, levels of hsa-miR-449a in HCC cell lines was enhanced significantly by inhibiting histone deacetylases (HDACs), which were up-regulated in HCC tissue (96). Reduced expression of miR-199a/b-3p, one of the consistently and markedly decreased miRNA in HCC, is mediated by histone methylation and independent of DNA methylation (97). On the other hand, some miRNAs have been reported to be involved in hepatocarcinogenesis by regulating histone deacetylases (HDACs), including miR-1, miR-22, and miR-200a targeting HDAC4, miR-31 and miR-145 targeting HDAC2, miR-221 targeting HDAC6, miR-29c targeting SIRT1, miR-125a-5p, and miR-125b targeting SIRT7, suggesting the potential use of miRNA-based therapies in HCC (98).

Chromatin modifiers or remodelers regulate accessibility to chromatin and positioning of nucleosome in the DNA. Upregulated enhancer of zeste homolog 2 (EZH2) in HCC, a well-studied chromatin modifier which mediates gene silencing in HCC, represses miR-622 by enhancing H3K27 trimethylation, and is correlated with unfavorable HCC prognosis (32). CCCTC-binding factor (CTCF) is a highly conserved insulator-binding protein with an enhancer-blocking function and contributes to the epigenetic regulation of some miRNAs (99). In breast cancer cells, disruption of CTCF binding at miR-125b1 CpG island (CGI) is associated with CGI methylation and the gain of the repressive histone marks including H3K9me3 and H3K27me3, and induces silencing of miR-125b1 expression (100). Considering the miR-125b is consistently down-regulated in HCC tissue (17, 30, 32) and serum (39, 40), disruption of CTCF binding might modulate HCC development.

Circular RNAs (circRNAs) are a class of highly conserved, stable and abundant non-coding RNAs (ncRNAs) that can regulate gene expression at transcriptional or post-transcriptional levels. The majority of circRNAs function as sponges of miRNA (101) and deregulation of a number of circRNAs have been reported in HCC. For example, circHIPK3 can sponge 9 miRNAs with 18 potential binding sites, including directly binding to the well-known tumor suppressor miR-124, reducing its activity (102). circTRIM33–12 acts as the sponge of miR-191 to suppress HCC (103). Artificial circRNAs which bind and sponge specific miRNAs can be constructed to achieve better inhibitory effects on oncogenic or pathogenic miRNAs, indicating a promising strategy to treat HCC.

## Regulating miRNA as a Therapeutic Approach for HCC

Normalization of dysregulated miRNAs in patients with HBV-HCC, by either up- or down-regulation of dysregulated miRNAs, is a plausible therapeutic approach in treating HCC.

Preliminary studies suggest that reestablishing the expression of down-regulated miRNAs might restore the tumor-suppressing function of miRNAs. In a first-in-human Phase 1 trial of a miRNA therapy using a liposomal miR-34a mimic in patients with advanced solid tumors including HBV-HCC, the miR-34a mimic showed antitumor activity (104). In another study upregulation of miR-122, which is frequently down-regulated in HCC patients, suppressed the proliferation and invasion capability of HCC-derived cells and increased sensitivity to chemotherapy (31, 105–107). Restoring miR-122 in stem-like HCC cells was shown to decrease cell proliferation and reduce tumor size in a mouse model (93). Besides miR-122, other miRNAs may have value in treating HCC. A recent study showed that injection of exosomal miR-335-5p, a tumor suppressor, can inhibit HCC cell proliferation and invasion as well as result in slower cancer growth (108). On the other hand, suppression of miR-21, which is frequently up-regulated in patients with HCC, leads to increased sensitivity to chemotherapeutic drugs (21).

In addition to direct targeting of miRNA, modulating the upstream genes that control miRNA expression is another therapeutic strategy. Upregulation of miR-122 by activating the farnesoid X receptor transcription factor (FXR), suppressed the proliferation of HCC cells *in vitro* and reduced the growth of HCC xenografts *in vivo* (109).

The crosstalk between epigenetics and miRNA related to HCC provides new opportunities for the development of more effective therapy for HCC by targeting epigenetic modulation of miRNAs as discussed above. Restoring the expression of tumor suppressor miRNA by inhibitors of DNA methylation and histone deacetylase, and inhibiting the expression of oncogenes by artificial circRNAs sponging specific miRNAs may be promising therapeutic strategies for HCC.

Regulating miRNA-mediated immune response in HCC may prove to be a promising therapeutic strategy. Most recently, Tian's group demonstrated that HBV mediates PD-L1-induced T cell immune exhaustion through the interaction of the oncofetal gene SALL4 and miR-200c (110). They showed that miR-200c controls PD-L1 expression by directly targeting the 3′-UTR of PD-L1 and that overexpression of miR-200c antagonizes HBV-mediated PD-L1 expression and reverses antiviral CD8^+^ T cell exhaustion.

A group of miRNAs are involved either directly or indirectly in drug resistance and either suppressing or activating miRNAs may reduce drug resistance. For example, a recent study reported that some miRNAs contribute to drug resistance to sorafenib. Targeting these miRNAs by the artificial long non-coding RNA improved treatment response in patients with HCC (111). Other studies found that restoration of miR-122 can sensitize HCC cancer cells to adriamycin and vincristine (112) as well as reverse doxorubicin-resistance in HCC cells (113). MiR-101 was shown to sensitize liver cancer cells to chemotherapeutic treatment (114).

The risk of undesirable effects of miRNA targeting, due in large part to off-target binding, is challenging. Adverse events were common in the miR-34a mimic trial, the first clinical trial for the treatment of HBV-HCC (104) and the trial was recently terminated due to immune-related serious adverse events (115). Of the clinical trials using miRNAs that are dysregulated in HBV-HCC, one phase II trial of miR-122 as a treatment modality for HCV has been completed and a miR-21 phase II trial for Alports syndrome was suspended (115). The application of miRNA-targeting therapy has strong potential in personalized medicine, although off-target effects remains a significant hurdle.

## miRNAs as Biomarkers in HBV-HCC

Early diagnosis of HCC, crucial for treatment outcome, remains challenging. The limitations of imaging technology and AFP detection to diagnose small and atypical HCC calls for more sensitive and specific biomarkers. Based on reports that many miRNAs are expressed differentially in HBV-HCC patients (Tables 1, 2) and miRNAs dysregulation is an early event in hepatocarcinogenesis occurring in pre-malignant dysplastic nodules (23, 47), the detection of miRNAs, especially circulating miRNAs levels, is gaining increasing recognition and attention for their potential clinical utility as biomarkers in screening and early diagnosis of HBV-HCC and predicting HCC prognosis as well.

### miRNAs as Diagnostic Biomarkers in HBV-HCC

Potential single miRNAs and miRNA panels that have been proposed as early diagnostic biomarkers for HBV-HCC are summarized in Table 4. Circulating miRNAs, including miR-18a, miR-21, miR-101, miR-122, miR-139, miR-223, and some miRNA panels may have diagnostic utility in distinguishing HBV-HCC patients from patients with chronic HBV infection (CHB) or liver cirrhosis (LC). Complicating the consensus and interpretation of the results of the studies (Table 4) are the differences in control groups employed [i.e., HBV-negative or HBV infected persons (CHB or LC) (22, 29, 42–46, 48, 116–118)].

**Table 4:** Diagnostic value of miRNAs in HBV-related HCC.

**miRNA**	**Sample details**				**Diagnostic value**				**References**
	**Sample type**	**Size of case**	**Underlying cirrhosis, %**	**Control**	**Specificity (%)**	**Sensitivity (%)**	**AUC**	**CI of AUC**	
miR-18a	S	101 HBV-HCC	NA	60 HC	75.0	86.1	0.881	0.829–0.933	(44)
		101 HBV-HCC	NA	30 CHB or LC	70.0	77.2	0.775	0.681–0.869	(44)
miR-21	S	101 HCC (76 HBV)	NA	89 HC	73.5	84	0.87	0.81–0.93	(42)
		57 HBV-HCC	NA	30 HC + 29 HBV	71.2	89.5	0.865	NA	(116)
miR-101	S	67 HBV-HCC	NA	30 HC	70.0	76.1	0.788	0.693–0.865	(29)
		67 HBV-HCC	NA	79 CHB	62.0	88.1	0.777	0.701–0.842	(29)
		67 HBV-HCC	NA	61 HBV-LC	90.2	95.5	0.976	0.931–0.995	(29)
miR-122	S	70 HBV-HCC	75% (51/68)	34 HC	83.3	81.6	0.869	0.786–0.952	(43)
		70 HBV-HCC	75% (51/68)	48 CHB	57.8	77.6	0.63	0.516–0.743	(43)
		101 HCC (76 HBV)	NA	89 HC	69.1	70.7	0.79	0.71–0.86	(42)
miR-139	P	31 HBV-HCC	NA	31 CHB	58.1	80.6	0.761	0.643–0.878	(22)
miR-223	S	101 HCC (76 HBV)	NA	89 HC	80	76.5	0.86	0.80–0.92	(42)
miR-15b and miR-130b	S	57 HBV-HCC	NA	30 HC + 29 HBV	91.5	98.3	0.981	NA	(116)
miR-27b-3p, miR-192-5p	S	212 HBV-HCC	NA	110 HC + 106 HBV- LC	91.2	68.6	0.836	0.783–0.880	(48)
				110 HC	95.2	68.5	0.823	0.748–0.866	
				106 HBV-LC	79.3	78.5	0.859	0.804–0.906	
miR-29a, miR-29c, miR-133a, miR-143, miR-145, miR-192, miR-505	S	153 HBV-HCC	NA	60 HC + 68 CHB + 71 HBV-LC	88.9	74.5	0.817	0.769–0.865	(117)
		153 HBV-HCC	NA	68 CHB + 71 HBV-LC	89.9	74.5	0.822	0.772–0.873	
		49 HBV-HCC	NA	48 HC + 42 inactive HBsAg carrier +	91.1	85.7	0.884	0.818–0.951	
		49 HBV-HCC	NA	42 inactive HBsAg carrier	83.3	85.7	0.845	0.758–0.932	
		27 HBV-HCC	NA	135 matched CHB	80.0	70.4	0.752	0.644–0.860	
miR-122, miR-192, miR-21, miR-223, miR-26a, miR-27a, miR-801	P	457 HBV-HCC	NA	141 HBV-LC + 169 CHB + 167 HC	83.5	81.8	0.888	0.852–0.917	(45)
				167 HC	93.9	83.2	0.941	0/905–0.966	
				169 CHB	76.4	79.1	0.842	0.792–0.883	
				141 HBV-LC	91.1	75	0.884	0.838–0.921	
miR122, miR1228, miR141, miR192, miR199a, miR206, miR26a, miR433	S	261 HBV-HCC	NA	173 HC + 233 HBV-LC	76.2	90.3	0.879	0.842–0.941	(46)
				173 HC	83.3	82.8	0.893	0.849–0.94	
				233 HBV-LC	84.6	81.6	0.892	0.844–0.939	
miR-20a-5p, miR-25-3p, miR-30a-5p, miR-92a-3p, miR-132-3p, miR-185-5p, miR-320a, miR-324-3p	P	67 HBV-HCC	NA	82 HBV	64.6	86.6	0.802	NA	(118)

*When data from training set and validation set are available, only the latter is presented. AUC, area under the curve; CHB, chronic hepatitis B; CI, confidence interval; HBV, Hepatitis B virus; HC, healthy control; HCC, hepatocellular carcinoma; LC, liver cirrhosis; NA, not available; P, plasma; S, serum*.

A recent study revealed that a seven-miRNA classifier (miR-29a, miR-29c, miR-133a, miR-143, miR-145, miR-192, and miR-505) had significantly higher sensitivity than AFP to discriminate between HCC and healthy controls, inactive HBsAg carriers, CHB patients, and HBV-cirrhosis patients. Critically this miRNA classifier was the first biomarker to diagnosis preclinical HCC, which was detected in eight of 27 HBV infected individuals 12 months before clinical diagnosis of HCC. This miRNA classifier holds promise for improving clinical outcomes by early HCC detection and curative treatment (117).

Among these miRNAs and miRNA panels, miR-122 is the most replicated miRNA biomarker in HCC, which has a sensitivity ranging from 71 to 81%, specificity from 59 to 83%, and an AUC from 0.63 to 0.87 to distinguish HBV-HCC from controls (42, 43). miR-122 is also included in two miRNA panels for HBV-HCC (45, 46). However, the diagnostic utility of miR-122 in HBV-HCC also extends to other HCCs (119).

Multiple approaches may be taken to improve the diagnostic performance of miRNA biomarkers in HBV-HCC. The type of biological sample is one of the key factors influencing sensitivity and specificity.

Exosomes are secreted by most cell types including cancer cells. Serum exosomes are highly enriched in miRNAs and exosomes can transfer miRNAs between cells, thus affecting HCC cancer proliferation, migration, metastasis, drug resistance (120). A meta-analysis published in 2019 suggested that exosomal miRNAs have superior diagnostic value in prostate cancer patients (121). With regard to diagnosis of HBV-HCC, recent studies indicate that exosomal miRNAs might also be a better choice than miRNAs from whole serum or plasma for early diagnosis. Wang et al. found that the detection of exosomal miR-21, which is enriched in exomes, had improved sensitivity over the whole serum (122). Similarly, miR-125b levels in exosomes were significantly lower than in serum from patients with HBV-HCC when compared to patients with CHB or LC, which explains, at least in part, why miR-125b levels in exosomes, but not in serum, independently predict HCC progression (40). Another study comparing HBV-HCC to CHB or LC, found a greater difference in miRNA levels in exosomes compared to whole serum (123). Combinations of miRNAs with other classic serum markers, i.e., AFP, is another approach to increase sensitivity and specificity of blood-based early detection of HBV-HCC (117, 118), especially for atypical HCC cases with lower serum AFP levels. The better performance of this add-on strategy was demonstrated in HCC cases caused by non-HBV factors as well (124).

### miRNAs as Biomarkers for HBV-HCC Prognosis

Expression levels of several miRNAs in liver tissue or circulation were correlated with disease severity and survival of HBV-HCC patients. Commonly reported single miRNAs and miRNA biomarker panels in predicting the survival of HBV-HCC are summarized in Table 5. Single miRNAs and miRNA panels associated with shorter survival include miR-21, miR-221, and two 20-mer miRNA signature profiles (20, 21, 25, 32, 47, 128, 129); miR-26a, miR-26b, miR-122, miR-125b, and miR-203 were associated with longer survival (27, 31, 40, 130). Among these miRNAs, miR-21 was the most replicated with a hazard ratio (HR) ranging from 1.4 to 2.2 in predicting the long-term progression of HBV-HCC (Table 5); miR-21 was also associated with HCCs (131). Given the enrichment of miRNAs in serum exosomes, detection of serum exosomal miRNAs can be used to predict prognosis of HCC patients (40).

**Table 5:** Prognostic value of miRNAs in HBV-related HCC.

**Sample**			**miRNA panels**	**Risk/protective** **factor**	**HR/RR**	**CI**	***P*-value**	**Outcome**	**References**
**Type**	**Size of case**	**Underlying cirrhosis, %**							
P/S	97 HCC (60 HBV)	32.0% (31/97)	miR-21	Risk	2.229	1.328–3.743	0.002	OS	(47)
P/S	136 HCC (129 HBV)	NA	miR-200a	Risk	1.75	1.45–2.11	<0.001	OS	(125)
P/S	62 HCC (40 HBV)	NA	miR-1246	Risk	10.24	1.39–75.67	0.023	OS	(126)
P/S	62 HCC (40 HBV)	NA	miR-1246	Risk	10.12	1.45–70.47	0.020	DFS	(126)
P/S	120 HBV-HCC	85.8% (103/120)	miR-26a	Protective	0.29	0.11–0.76	0.011	LT-free survival	(127)
P/S	120 HBV-HCC	85.8% (103/120)	miR-29a	Protective	0.36	0.15–0.91	0.030	LT-free survival	(127)
Exosome	128 HCC (121 HBV)	76% (97/128)	miR-125b	Protective	0.36	0.18–0.74	0.005	OS	(40)
T	148 HCC (82 HBV)	41% (45/109)	miR-21	Risk	NA	1.19–1.47	0.004	DFS	(21)
T	140 HBV-HCC	NA	miR-21	Risk	1.509	1.079–2.112	0.016	3-years OS	(128)
T	108 HBV-HCC	NA	miR-21	Risk	1.416	1.057–1.897	0.020	5-years OS	(128)
T	115 HCC (101 HBV)	51% (59/115)	miR-221	Risk	2.09	1.09–4.04	0.027	MFS	(20)
T	135 HCC (96 HBV)	95% (128/135)	miR-221	Risk	2.846	1.564–5.181	0.001	DFS	(25)
T	135 HCC (96 HBV)	95% (128/135)	miR-221	Risk	2.969	1.629–5.408	<0.001	OS	(25)
T	166 HCC (146 HBV)	84% (139/166)	20-miRNA prognostic signature^*^	Risk	2.75	1.58–4.79	<0.001	OS	(129)
T	214 HBV-HCC	93% (199/214)	20-miRNA metastasis signature^#^	Risk	2.1	1.2–3.6	0.01	OS	(32)
T	455 HCC (412 HBV)	88% (400/455)	miR-26a	Protective	0.48	0.21–1.0	0.05	OS	(27)
T	455 HCC (412 HBV)	88% (400/455)	miR-26b	Protective	0.48	0.20–0.91	0.04	OS	(27)
T	142 HCC (103 HBV)	58% (82/142)	miR-122	Protective	NA	NA	0.001	OS	(31)
T	120 HCC (97 HBV)	78% (93/120)	miR-200a	Protective	0.382	0.215–0.896	0.004	OS	(33)
T	101 HCC (71 HBV)	NA	miR-200a	Protective	0.403	0.242–0.670	<0.001	OS	(34)
T	66 HCC (64 HBV)	NA	miR-203	Protective	0.202	0.064–0.638	0.006	RFS	(130)
T	66 HCC (64 HBV)	NA	miR-203	Protective	0.332	0.139–0.793	0.013	OS	(130)

* miR-708, miR-34c-3p, miR-584, miR-4310, miR-744, miR-141, let-7d, miR-15a, miR-142-3p, miR-10b, let-7e, miR-28-3p, miR-193b, miR-101, miR-451, miR-142-5p, miR-26b, miR-497, miR-29c, miR-140-3p.

# miR-338, miR-219-1, miR-207, miR-185, miR-30c-1, miR-1-2, miR-34a, miR-19a, miR-148a, miR-124a-2, miR-9-2, miR-148b, miR-122a, miR-125b-2, miR-194, miR-30a, miR-126, let-7g, miR-15a, miR-30e.

*CI, confidence interval; DFS, disease-free survival; HBV, Hepatitis B virus; HR, hazard ratio; HCC, hepatocellular carcinoma; LT, liver transplantation; MFS, metastasis-free survival; OS, overall survival; P/S, plasma or serum; Ref, reference; RFS, recurrence-free survival; RR, relative risk; T, tissue*.

It should also be noted that other studies found no significant associations with survival between HBV-HCC patients with high or low levels of miRNAs, (i.e., miR-21, miR-122, and miR-125b) (126, 132, 133). These disparate results may be due to differences in study design, analysis, and participant characteristics. For example, the cut-off value used to divide high and low miRNA-expressed population varies among studies and can be quite arbitrary [e.g., using a fixed value or average value or optimal cut-off value from Youden index analysis, or a ratio comparison to adjacent non-tumor tissue] (20, 21, 40, 126). The outcome events also varied, including overall survival (OS), disease-free survival (DFS), recurrence-free survival (RFS), and liver transplantation (LT)-free survival. These differences among studies make comparison challenging. These limitations will need to be addressed to establish reliable diagnostic and prognostic miRNA biomarker panels for HBV-HCC.

## Dysregulated miRNAs in HCV-HCC

Since effective HCV-curative, direct-acting antiviral agents (DAA) are widely used worldwide in recent years (134, 135), fewer cases of HCC will be caused by HCV infection in the future. Subsequently, HBV infection will likely be the predominant cause of HCC worldwide. The pattern of dysregulated miRNAs in HCV-HCC, nevertheless, may still shed insights on the HBV-HCC pathogenesis as the comparison may reveal pathogen-specific and pathogen-independent tumorigenic pathways.

Several miRNAs showed similar dysregulation patterns in HCV- HCC and HBV-HCC (Table 6), including up-regulation of miR-18 (136), miR-221 (137) and miR-224 (15, 138, 139), and down-regulation of miR-199a-5p (136). These miRNAs may be involved in key cancer pathways that are shared by HBV- and HCV-HCC, including the WNT/β-Catenin and TP53 pathways. These miRNA and pathways may, therefore, be putative common targets for diagnostic, prognostic, and therapeutic interventions. Direct comparisons of miRNAs in HBV- and HCV-HCCs are lacking. In a small study comparing HBV-HCC and HCV-HCC tumor samples, the abundance of miR-122 was significantly reduced in HBV-HCC but not HCV-HCC, providing evidence of pathogen-specific dysregulation of miRNAs (74).

**Table 6:** Common microRNAs dysregulated consistently in HBV-HCC and HCV-HCC.

**Dysregulation type**	**miRNAs**	**Publications in HBV-HCC**	**Publications in HCV-HCC**
Up-regulated	miR-18a	(17, 18, 44)	(136)
	miR-221	(17, 19, 20, 22, 23, 25, 41)	(137)
	miR-224	(17, 18, 23, 41)	(15, 138, 139)
Down-regulated	miR-199a-5p	(19)	(136)

## Challenges and Future Directions

Accumulating evidence indicates that miRNAs, which function as gene regulators at the post-transcriptional level, are involved in the development of HBV-HCC. The expression levels of some single miRNAs or miRNA panels have the specificity and sensitivity to diagnose HCC and to predict survival; therefore, miRNA profiling panels are promising biomarkers for early diagnosis and survival prediction of HCC (Figure 1). Clinical trials to establish the utility of these panels in clinical practice are warranted.

However, there are several limitations and knowledge gaps in the current literature. In HBV-HCC, most HCC arise from cirrhotic tissues, thus miRNA changes may originate from either or both HCC and cirrhotic tissues. Underlying cirrhosis was present in 45–95% of HCC cases among studies that reported this information (Table 1), other studies did not report cirrhosis status. How miRNA profiles differ between cirrhotic and non-cirrhotic HBV-HCC remains largely unexplored (140).

The heterogeneity of methodologies in control selection, miRNA detecting technologies, case and control characteristics, and biostatistical analyses in studies also contribute to different results among studies. Failure to replicate findings may be due to small sample size affecting power leading to type 1 and type 2 errors. A major confounder among the studies is the selection of control tissue or sample. For example, comparisons may be made between tumor and non-tumor tissues from the same patients or different individuals. qRT-PCR quantification methods and platforms for miRNAs vary in their sensitivity and breadth. Technical replication to control for between and within-sample variation was lacking in some studies (42). Although most studies use internal controls to normalize miRNA expression levels of target genes (e.g., U6 SnRNA, GAPDH, miR-16, RNU43, cel-miRNA-39, or synthetic cel-miR-67), no universal internal references are used making comparisons among studies challenging (32, 36, 42, 47, 117, 122, 141, 142). Reviewers and journals are aware that a lack of replication in clinical research is a growing area of concern. A common set of internal controls would facilitate the replication and validation of informative miRNAs. Another source of failure to replicate is that the coverage of the miRNA arrays varies by more than 2-fold (308 to 829 miRNAs) (17–19, 32, 45). Definitions of differential expression vary from >2-fold change to <1.5 change in others. Over conservative cut-offs tend to lead to type 2 errors while less conservative cut-offs tend to increase type 1 errors. Next-generation sequencing is particularly prone to mis-annotations of microRNAs, which may lead to false-positive (143) or false-negative findings (144).

Before miRNAs can be used in a clinical setting, standardized methods for sample collection and handling should be implemented. Clinical trials will need to be conducted to assess the performance of miRNA biomarkers in addition to or in place of current diagnostic methods before their acceptance into surveillance or screening programs or for clinical management of HCC. We consider design issues and knowledge gaps that warrant attention in future investigations.

1. Sample size: is a major factor affecting power and validity. Since most miRNA have a moderate (<3-fold) difference between cases and controls and both large intra-individual and inter-individual variation, large sample sizes are required for sufficient power to minimize type 1 and II errors. Replication using public datasets [e.g., the Cancer Genome Atlas (TCGA) database] may provide additional supporting evidence (145, 146).
2. Validation for circulating miRNAs: To develop liquid biopsies for detection, diagnosis, and prognosis, miRNAs identified from serum/plasma should be validated to miRNAs obtained from tumor tissue before clinical evaluation as biomarkers. Non-specific circulating miRNAs may originate from other high blood-flow organs and tissue (147).
3. Clinical trials: Promising miRNAs markers must be tested for efficacy vs. standard of care (imaging and AFP levels) in randomized clinical trials before entering clinical practice.
4. HCC early detection: Since HCC is usually diagnosed mid to late-stage HCC, early HCC is rarely studied for miRNAs. Data comparing miRNAs expression levels in LC and early HCC groups is scarce and is urgently needed, as most HBV-HCCs develop from cirrhotic liver tissue. Clinical trials for miRNA early-diagnosis should focus on patients with HBV, HCV, or liver cirrhosis at high risk for HCC.
5. miRNA profiling for HBV-HCC: Evaluation of differences and commonalities of miRNA profiles in HCCs arising from HBV and other underlying liver diseases
6. Personalized medicine: Basic and clinical investigations for the clinical utility of precision miRNA-targeting therapies.
7. Diversity of miRNA investigations: Most HBV-HCC studies have enrolled Asian patients because of their high carrier rate for HBV. However, it is unknown if miRNA results are similar across diverse populations, particularly in Africa where HBV prevalence is also high (73). The generalizability of findings in Asians needs to be tested in other global populations.

Taken together, the recent studies in miRNAs provide encouraging evidence that miRNAs detection may aid in the diagnosis, survival prediction, and treatment of HBV-HCC. More well-designed and well-powered case-control or longitudinal studies in diverse populations are critically needed to validate the utility of miRNAs in HCC and translate miRNA into clinical use.

## Author Contributions

JX and PA conceived idea, prepared the tables, and wrote the manuscript. YY and CW revised the manuscript. All authors read and approved the final manuscript.

## Conflict of Interest

The authors declare that the research was conducted in the absence of any commercial or financial relationships that could be construed as a potential conflict of interest.

## Abbreviations

3′ UTR: 3′ untranslated region
AFP: alpha-fetoprotein
ANT: adjacent non-cancerous tissue
ASC: asymptomatic carrier
API-5: apoptosis inhibitor-5
AUC: area under curve
CCNG1: cyclin G1
CGI: CpG island
CHB: chronic hepatitis B
CI: confidence interval
CTCF: CCCTC-binding factor
CTGF: connective tissue growth factor
DFS: disease-free survival
DN: dysplastic nodules
EMT: epithelial-mesenchymal transition
ERα: estrogen receptorα
EZH2: Enhancer of zeste homolog 2
FXR: farnesoid X receptor
HBV: hepatitis B virus
HBV-HCC: HBV-related HCC
HBV-LC: HBV-related LC
HBx: HBV X protein
HC: healthy control
HCC: hepatocellular carcinoma
HCV: hepatitis C virus
HDAC: histone deacetylases
HGDN: high-grade dysplastic nodule
HR: hazard ratio
JAK/STAT: Janus kinase/signal transducer
LC: liver cirrhosis
LGDN: low-grade dysplastic nodule
LT: liver transplantation
miRNAs: microRNAs
mRNAs: messenger RNAs
NA: not available
NBNC: non-HBV non-HCV
onco-miRNA: oncogenic miRNA
OR: odds ratio
OS: overall survival
*PBF*: pituitary tumor-transforming gene 1 binding factor
*PTTG1*: pituitary tumor-transforming gene 1
P: plasma
P/S: plasma or serum
PI3K/MAPK: phosphoinositide 3-kinase/mitogen-activated protein kinase
RFS: recurrence-free survival
ROC: receiver-operator characteristic curve
RFS: recurrence-free survival
RR: relative risk
S: serum
T: tissue
WNT/β-Catenin: wingless-related integration site/beta-catenin.
